# Fluoride release and flexural strength of four ion-releasing restorative materials: An in vitro comparative study

**DOI:** 10.4317/jced.61818

**Published:** 2024-10-01

**Authors:** Patricia Llancari-Alonzo, Daniel Alvítez-Temoche, Marysela Ladera-Castañeda, Leonor Castro-Ramirez, Carlos López-Gurreonero, César Cayo-Rojas

**Affiliations:** 1 Universidad Nacional Federico Villarreal, Faculty of Dentistry, Lima, Peru; 2 Universidad Privada San Juan Bautista, School of Stomatology, Lima, Peru

## Abstract

**Background:**

This study aimed to determine the fluoride release and flexural strength of four ion-releasing restorative materials.

**Material and Methods:**

A total of 80 samples of four different materials were prepared in standardized molds: Ketac Universal, Beautifil II, Cention N, and Equia Forte Fil. The fluoride release was quantified at 1, 3, 7, 14, and 28 days post-immersion using an ion-selective electrode. The flexural strength was measured with a universal testing machine after 7 days of immersion in deionized water. A one-factor intergroup ANOVA with Welch’s robust variance and Games-Howell’s post hoc was employed. To compare related measures, a Friedman test with Bonferroni’s adjusted post hoc was employed. The *p*-value was set at 0.05.

**Results:**

At 7 days, significant differences were observed in the flexural strength of the four ion-releasing restorative materials (*p*<0.001). The flexural strength values from highest to lowest were as follows: Cention N (97.10 ± 4.99 MPa), Beautifil (82.77 ± 5.30 MPa), Equia Forte Fil (31.38 ± 7.68 MPa), and Ketac Universal (19.23 ± 2.94 MPa). In addition, at 3 and 7 days, the highest amount of fluoride released was observed for Cention N compared to the other ion-releasing restorative materials (*p*<0.05). Conversely, Beautifil II released the lowest amount of fluoride at 3, 7, 14, and 28 days (*p*<0.05) compared to the other ion-releasing restorative materials.

**Conclusions:**

The immersion of all ion-releasing restorative materials in deionized water for seven days resulted in significant differences in flexural strength. Cention N had the highest value, while Ketac Universal had the lowest. All ion-releasing restorative materials released fluoride at all test times, with Ketac Universal releasing the most at 1, 14, and 28 days, and Cention N releasing it at 3 and 7 days. Beautifil II showed the lowest fluoride release at all evaluated times, exhibiting a nearly constant release over time compared to the other materials.

** Key words:**Fluoride release, flexural strength, glass ionomer.

## Introduction

Dental caries is a chronic, multifactorial, non-communicable infectious disease mediated by oral biofilm and modified by diet (1,2). The cariogenic bacteria in the biofilm break down sugars and make acids that attack the tooth’s hard tissues, wearing them down over time and creating a carious lesion (3,4). The prevalence of dental caries remains high, and significant challenges remain in its prevention and treatment (5). As a result, the paradigm shift in how we approach these lesions has led to the development of new restorative materials.

The goal of minimal intervention dentistry for the management of carious lesions is to identify these lesions early, remineralize them, and manage active lesions in either enamel or dentin using both surgical and non-surgical technique (6,7). Furthermore, a personalized risk analysis for each patient will guide the treatment selection (8,9). This philosophy prioritizes the preservation of tooth structure and the concept of repairing rather than restoring to halt the progression of carious lesions (6,7). For this type of repair, remineralizing treatments of the remaining structure are essential (6,10).

Because resins have a relatively high incidence of failure as a result of secondary caries and bond deterioration at the interface, they are typically subject to frequent replacement (11,12). The recognition of these deficiencies led to the search for materials that would provide additional benefits for maintaining dental health. This resulted in the development of ion-releasing restorative materials that play a dynamic role in the oral cavity (13,14). Ion-releasing restorative materials can mimic physiological mineralization mechanisms and restore the tissue’s usual mechanical properties, allowing for complete recovery (6). Furthermore, it should be capable of releasing fluoride over an extended period, particularly in patients with a moderate to high risk of dental caries (15).

Nevertheless, these ion-releasing restorative material exhibit certain drawbacks, including poor hydrolytic stability, low flexural strength, and toughness. Furthermore, the presence of reactive fillers results in a decrease in the materials’ physical and mechanical properties, which can compromise the strength and longevity of the restorations. Additionally, this can lead to a rapid decrease in fluoride release (16,17).

The first ion-releasing restorative material to enter the market were conventional glass ionomers (GICs). Researchers have demonstrated that their fluoride release aids in preventing demineralization and promoting remineralization (18). However, their strong tendency to abrasion and fracture (19) prompted the development of improved formulations known as high-viscosity glass ionomer cements (HV-GIC), which promise superior wear resistance. Also, these ionomers might have extra resinous material in them, like RM-GIC (resin-modified glass ionomer cement), which has better flexural properties (19,20).

In contrast, a recently introduced material, designated “Alkasite”, has emerged on the market. This material is capable of releasing ions and fluorides that neutralize acids, in addition to exhibiting favorable surface hardness (19,20).

The existing literature lacks data regarding the flexural strength of these novel, advanced fluoride-releasing restorative materials. This study aimed to compare the flexural strength and fluoride release of four ion-releasing restorative materials *in vitro*. There would be no significant differences in the amount of fluoride released or the flexural strength of the ion-releasing restorative materials under investigation, according to the null hypothesis.

## Material and Methods

-Study design

This prospective, longitudinal, and experimental *in vitro* study was conducted at the Oral Health Research Laboratory (LISO) of the Universidad Peruana Cayetano Heredia and the High Technology Laboratory Certificate, Lima, Peru, from June to August 2023. In addition, this study adhered to the guidelines set forth by the Checklist for Reporting In-Vitro Studies (CRIS) (22).

-Ethical considerations

This study was approved by the Ethics Committee of the Faculty of Dentistry of the Universidad Nacional Federico Villarreal (UNFV) with letter No. 007-01-2023 dated January 16, 2023.

-Sample Calculation and Selection

**Figure 1:**
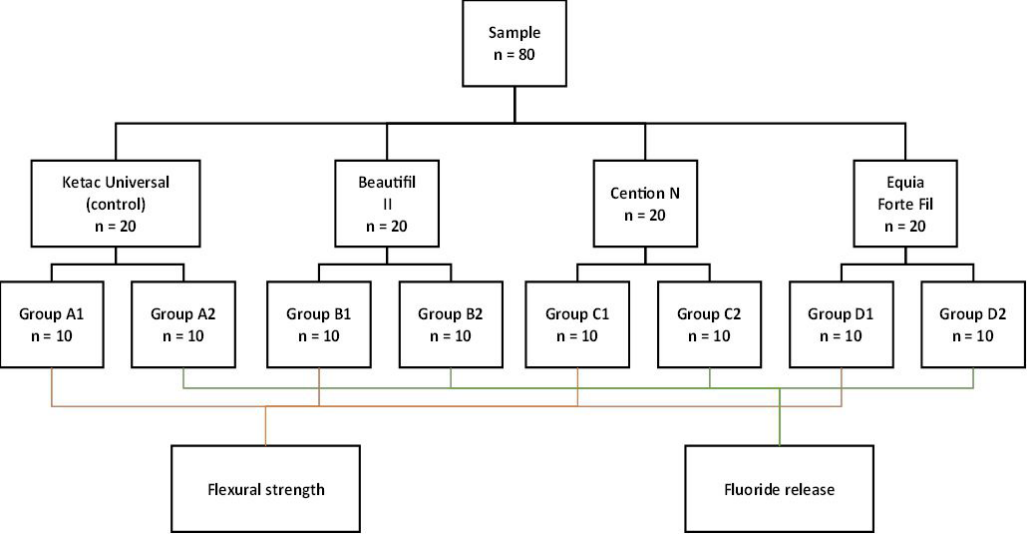
Distribution of groups randomly according to the type of material used. Flexural strength was evaluated in groups A1, B1, C1, and D1. Fluoride release was evaluated in groups A2, B2, C2, and D2.

The total sample size (n = 80) was calculated based on the data obtained in a pilot study conducted prior to the final experiment. The calculation was conducted using the formula for analysis of variance (ANOVA) within the statistical software G*Power (version 3.1.9.2). A significance level (α) of 0.05, statistical power (1-β) of 0.80, and an effect size of 9.86 were employed with four independent groups involved. A total of 80 samples were then fabricated, standardized, and randomly distributed into four groups of 20 samples each. Subsequently, the groups were randomly divided into two equal subgroups, according to the flexural strength test (n = 10) and fluoride release (n = 10) (Fig. 1).

-Sample preparation

Samples were prepared using the following materials: Ketac Universal (3M/ESPE, St. Paul, MN, USA), Beautfil II (Shofu Inc., Kyoto, Japan), Cention N (Ivoclar Vivadent, Schaan, Liechtenstein), and Equia Forte Fil (GC Corporation, Tokyo, Japan).

Ketac Universal and Cention N were properly mixed on a waxed paper pad using a plastic spatula. The powder-to-liquid ratios utilized and the other procedures were conducted in accordance with the manufacturer’s instructions. The samples were then allowed to self-cure in accordance with the instructions provided. Subsequently, Cention N was subjected to light curing with an LED lamp (Valo®, Ultradent, South Jordan, Utah) at 1000 mW/cm2 for 20 seconds.

Beautifil II was placed directly onto the mold and light-cured with an LED lamp (Valo®, Ultradent South Jordan, Utah) at 1000 mW/cm2 for 20 seconds.

Equia Forte Fil was prepared with a Silver Mix capsule mixer (GC Corporation, Tokyo, Japan) for 10 seconds in accordance with the manufacturer’s instructions. Subsequently, the material was placed in a mold using a capsule dispenser. Equia Forte Coat was applied and light-cured with a LED lamp (Valo®, Ultradent South Jordan, Utah) at 1000 mW/cm2 for 20 seconds.

The intensity of the light curing unit was evaluated using a radiometer (Woodpecker® LM-1, Woodpecker, Guilin, Guangxi, China).

All samples were coated with a strip of celluloid and gently pressed with the aid of a glass plate to remove any excess material.

To assess the flexural strength, 10 samples (n = 10) were prepared for each restorative material using metal molds with dimensions of 2 mm width × 2 mm depth × 25 mm length (15,17,23,24). Subsequently, the samples were stored at 37°C for 24 hours to permit complete polymerization (15,17,23).

To assess the release of fluoride, ten disks (n = 10) were made for each type of restorative material using stainless steel molds that were 6 mm in diameter and 4 mm thick (25). Following the initial setting period, the samples were placed in test tubes with tight caps to prevent the solution from evaporating and stored in deionized water (5 mL) at 37°C ± 2 °C (25-27). At 24-hour intervals, the samples were transferred to 5 mL of deionized water.

-Flexural strength test

**Figure 2:**
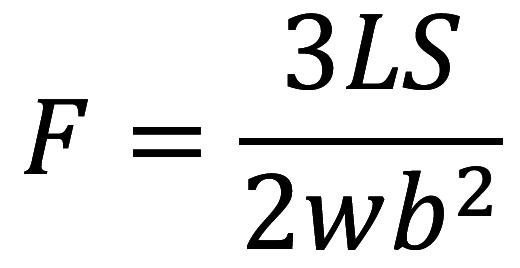
Formula.

On the seventh day of immersion in deionized water, a three-point flexural test with a length of 20 mm was applied at a crosshead speed of 1 mm/min using a computer-controlled universal testing machine (INSTRON 5965 loading frame, Boston, MA, USA) (13,15). The following formula was employed, (Fig. 2):

Where F = flexural strength, L = maximum load (N), S = distance between supports (mm), w = sample width (mm), and b = sample height (mm).

-Fluoride release test 

Measurements were conducted using a fluoride ion-selective electrode (Orion 9609BNWP, Ionplus Sure-Flow Fluoride, Thermo Scientific, USA) coupled to a benchtop analyzer (Orion Star™ series ISE meter, Thermo Scientific, Beverly, USA) with a detection limit of ± 0.001 ppm (parts per million) (26). The electrode was calibrated with a standard fluoride solution with concentrations of 0.1, 1, 10, and 100 ppm (29,30). Once a suitable calibration curve was observed, the readings were taken.

To quantify the fluoride concentration, 5 mL of TISAB II solution (Total Ionic Strength Adjustment Buffer; Merck KGaA, Darmstadt, Germany) was added to the 5 mL solution of deionized water in which the sample was immersed (26,29,31,32). Subsequently, the fluoride ion-selective electrode was introduced into the test tube, and the fluoride concentration in parts per million (ppm) was recorded (29,32).

Once the fluoride concentration readings had been obtained, each sample underwent a thorough rinsing with deionized water. It was then placed back into clean, tightly sealed test tubes for storage at a temperature of 37°C until the next measurement could be taken (29,30,32).

All data (ppm) were recorded at intervals of 1, 3, 7, 14, and 28 days.

-Statistical analysis

The data were subjected to statistical analysis using SPSS v28.0 software. For the purposes of descriptive analysis, measures of central tendency, such as the mean and median, were calculated. The standard deviation and interquartile range were employed to calculate measures of dispersion. For hypothesis testing regarding flexural strength (in MPa) and fluoride release (in ppm), an intergroup one-factor ANOVA with Welch’s robust variance and Games Howell’s post hoc test was employed. To compare measures related to fluoride release, the Friedman test with Bonferroni-adjusted post hoc was employed. Prior to selecting the appropriate statistical test, the data were subjected to a Shapiro-Wilk test to ascertain their normal distribution and a Levene’s test to assess homoscedasticity. For all statistical analyses, the significance level was set at *p* <0.05.

## Results

The average flexural strength (MPa) of Cention N (Alkasite) was the highest of the four ion-releasing restorative materials with 97.10 MPa (95% CI: 93.53 MPa–100.66 MPa), while the average for Ketac Universal (GIC) was the lowest at 19.23 MPa (95% CI: 17.12 MPa–21.33 MPa). It was also discovered that Cention N (Alkasite) had a significantly higher flexural strength than Beautifil II (Giomer) (*p* < 0.001), which in turn had a significantly higher flexural strength than Equia Forte Fil (HV-GIC) (*p* < 0.001), and that last one had a significantly higher flexural strength than Ketac Universal (GIC) (*p* < 0.001) ( Table 1).

**Figure 3:**
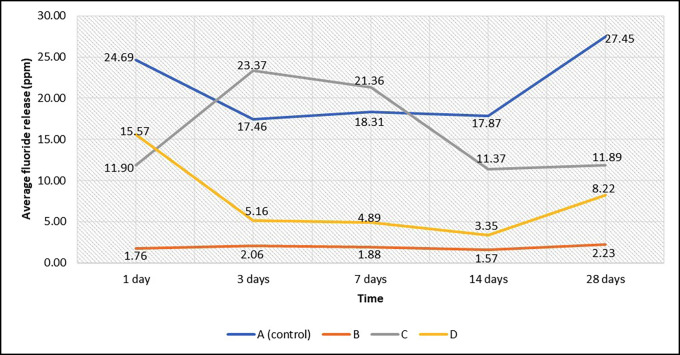
Average fluoride release (ppm) of ion-releasing restorative materials over time. A: Ketac Universal (GIC), B: Beautifil II (Giomer), C: Cention N (Alkasite), D: Equia Forte Fil (HV-GIC).

Significant variations in fluoride release (ppm) were observed in all ion-releasing restorative materials over time (*p*<0.05). In Ketac Universal (ionomer), a fluoride release of 24.69 ± 0.94 ppm in deionized water was seen on day 1. On day 3, it dropped significantly and reached its lowest level of 17.46 ± 0.79 ppm. On day 7, there was a significant increase (*p* = 0.047), which stayed the same until day 17 (*p* = 1.000). Finally, on day 28, there was a significant increase (*p*<0.001) with a fluoride release of 27.45 ± 0.84 ppm, which was the highest average ever recorded. On day 1, Beautifil II (Giomer) released 1.76 ± 0.27 ppm fluoride. This level increased significantly on day 3 (*p* = 0.030) and stayed the same until day 7 (*p*= 1.000). It then decreased significantly on day 14 (*p* = 0.047), reaching its lowest average of 1.57 ± 0.24 ppm. Finally, it increased significantly on day 28 (*p*<0.001), reaching its highest average of 2.23 ± 0.19 ppm fluoride. On day 1, Cention N (Alkasite) released 11.90 ± 1.11 ppm of fluoride. This level significantly increased on day 3 (*p* = 0.002) to an average of 23.37 ± 1.21 ppm, which was the highest recorded level. It stayed the same until day 7 (*p* = 1.000), then dropped significantly on day 14 (*p* = 0.001) to an average of 11.37 ± 0.94 ppm, which was the lowest recorded level. It stayed the same until day 28 (*p* = 1.000). Lastly, Equia Forte Fil (HV-GIC) had its highest average on day 1 with a value of 15.57 ± 0.73 ppm, then dropped significantly on day 3 (*p* = 0.047) and didn’t change significantly again until day 7 (*p* = 1.000). Its lowest average was on day 14 with 3.35 ± 0.47 ppm, and it increased significantly on day 28 (*p*<0.001) with a value of 8.22 ± 0.58 ppm fluoride ( Table 2, Fig. 3).

When comparing the ion-releasing restorative materials on day 1, Ketac Universal (GIC) released significantly more fluoride in ppm than Equia Forte Fil (HV-GIC), which released significantly more than Cention N (Alkasite), and the latter released significantly more than Beautifil II (Giomer) (*p* 0.001, *p* 0.001, *p* 0.001, and *p* 0.001, respectively). On days 3 and 7, Cention N (Alkasite) released significantly more fluoride than Ketac Universal (GIC), which released significantly more fluoride than Equia Forte Fil (HV-GIC), and the latter released significantly more fluoride than Beautifil II (Giomer) (*p*<0.001, *p*<0.001, *p*<0.001, and *p*<0.001, respectively). Finally, at 14 and 28 days, Ketac Universal (GIC) released significantly more fluoride than Cention N (Alkasite), which released significantly more fluoride than Equia Forte Fil (HV-GIC), and the latter released significantly more fluoride than Beautifil II (Giomer) (*p*<0.001, *p*<0.001, *p*<0.001, and *p*<0.001, respectively) ( Table 3).

## Discussion

In order to reduce the susceptibility of resin composites to secondary caries, ion-releasing restorative materials have been developed. Such materials are capable of releasing fluoride over time; thus, they must possess adequate flexural strength (15). However, the presence of reactive fillers has been shown to result in a reduction in the physical and mechanical properties of the material, which could potentially lead to a rapid decrease in fluoride release. This has been evidenced in studies where the use of reactive fillers has been linked to a reduction in the fluoride release rate (16,17). The present study aimed to compare the flexural strength and fluoride release of four ion-releasing restorative materials *in vitro*. Cention N, Beautifil II, Equia Forte Fil, and Ketac Universal showed variable flexural strength values with significant variations in fluoride release (ppm) at days 1, 3, 7, 14, and 28, rejecting the null hypothesis.

Flexural strength tests are important because they measure the oral cavity’s occlusal forces. This study revealed significant differences between the four materials, with Cention N demonstrating higher values, followed by Beautifil II, Equia Forte Fil, and Ketac Universal. It was the same as what Vandana *et al*. (15), François *et al*. (19), Battula *et al*. (33), and Balagopal *et al*. (34) found: Cention N had higher flexural strength values than other materials like GIC, HV-GIC, and RM-GIC from different commercial houses. This could be due to the fact that Cention N is an alkasite and has a different composition than ionomer-based materials. It has an organic monomer in the liquid that is made up of four different dimethacrylates: urethane dimethacrylate (UDMA), tricyclodecanedimethanol dimethacrylate (DCP), and polyethylene glycol 400 dimethacrylate (PEG-400 DMA). It has been demonstrated that these dimethacrylates undergo a change in chemical composition during the polymerization reaction (15,35). UDMA is the principal component of the monomeric matrix and is deemed to be the primary factor responsible for the elevated strength values, as postulated by numerous authors (19,33,34). Furthermore, its higher viscosity and the absence of hydrophobic hydroxyl side groups, which exhibit lower water absorption, contribute to its superior mechanical properties (15). The cyclic aliphatic structure of DCP facilitates the enhancement of strength. The powdered material contains a variety of fillers, including barium aluminum silicate glass filler, ytterbium trifluoride, isofiller (Tetric N-Ceram technology), calcium fluorosilicate glass filler, barium, aluminum, and calcium fluorosilicate (15,36,37). The particle sizes of these fillers range from 0.1 nm to 35 nm, which is responsible for providing adequate strength. Furthermore, isofillers are patented fillers that are mixed with silanes, which adhere to other particles and serve to reinforce the bond between the organic monomer matrix and the inorganic filler (15).

The findings of Hiremath *et al*. (35), Marovic (37) and Moshaverinia *et al*. (38) are inconsistent with those of the present study, as they report higher flexural strength values for composite resin and RM-GIC materials compared to Cention N. It is noteworthy that Cention N was not light-cured in these studies. This resulted in the redox polymerization commencing at a later point in the self-curing mode, occurring between 3.5 and 11 minutes later than it did with the same light-cured material. This resulted in a change to the degree of conversion and the material’s properties. It would be inadvisable to utilize this material without supplementary light curing. This finding is consistent with the results of studies conducted by Gomes de Arajo *et al*. (39) and Aldhafyan *et al*. (40), which demonstrated a significantly lower degree of conversion in self-curing materials compared to light-curing materials when dual curing was employed.

The ability to release fluoride depends on the material’s charge content and the nature of the glass-ionomer hydrogel matrix phase (26,42). Fluoride release is initially high and gradually decreases over time. This has been called the “explosion effect” and is normal for glass ionomers (26,28,29,32). This study evaluated two ionomers, Ketac Universal (GIC) and Equia Forte Fil (HV-GIC), which released high amounts of fluoride during the first 24 hours and then showed a decrease, consistent with the aforementioned effect and in line with reports from various authors (26,28,29,32). The initial acid-base reaction between glass and polyalkenoic acid releases fluoride that is weakly attached to the glass ionomer cement. Subsequently, a gradual release occurs, which compensates for the erosive leaching of glass particles within the cement and facilitates the dissemination of the leached fluoride throughout the cement matrix (26,29,32). The explosive effect is of significance for the process of remineralization and the reduction of the viability of any microorganisms that may have remained in the carious dentin (29,32).

Over time, the Beautifil II giomer did not demonstrate a burst effect, but rather a low and consistent fluoride release. This finding is consistent with the conclusions of Dasgupta *et al*. (26) and Dhumal *et al*. (43). The discrepancy may be attributed to the composition of the material, which contains a pre-reacted glass ionomer (S-PRG). This is formed by pre-reacting fluoroaluminosilicate glass with acid prior to its incorporation into the resin matrix. Consequently, the fluoride-containing glass has a limited hydrogel matrix phase (26,43,44). Moreover, the presence of resins such as UDMA could result in the hydrolytic disintegration of bioglass particles (28). Another factor that affects the release of fluoride is the porosity of the material, which influences the solubility of the filler. The addition of resin monomers to Beautifil II’s composition results in a lower porosity than that observed in GIC and HV-GIV. This results in the formation of a higher diffusion barrier, which impedes the passage of fluoride and water (28,43,44).

Cention N exhibited a moderately low fluoride release that increased over time, which was consistent with the findings of Banic *et al*. (31), Balogopal *et al*. (34), and Feiz *et al*. (45). Cention N’s alkasite nature accounts for its low initial fluoride release rate. In this case, the surface of the fillers changes, making them more resistant to breaking down and possibly reducing the release of fluoride ions (31,33,45). This suggests that the material matrix must undergo a sufficient degree of maturation over a defined period of time in order to facilitate the release of fluoride (31,33). The third and seventh days of immersion in deionized water serve to illustrate this point. The altered surface of the Cention N filler materials renders them resistant to degradation, which may result in the release of small quantities of fluoride ions (45).

Reactive filler can reduce the physical and mechanical properties of glass ionomers, potentially leading to a rapid decrease in fluoride release (16,17). Equia Forte Fill and Ketac Universal have demonstrated this phenomenon, displaying low flexural strength values and reduced fluoride release on the seventh day. These materials, being glass ionomers, exhibit the “explosion effect” (26,28,29,32), which leads to the erosive leaching of the glass particles (26,29,32), thereby generating a degradation mechanism in the material’s composition and compromising its flexural strength. But this didn’t happen with Cention N (alkasite) and Beautifil II (giomer), which gave off more fluoride and had the strongest flexural strength on the third and seventh days. Their different composition from GICs could explain their variable fluoride release mechanism and lack of the “explosion effect.” Additionally, they contain a thick polymer network that provides greater resistance to degradation (26,31). Conversely, some authors have posited that a specific period of time is necessary for the matrix of the materials to mature and present optimal properties (29,32,36). Based on the results, this requirement would only need to be met by ion-releasing restorative materials like alkasite and glyomers. However, it would not be a requirement for GICs, as they would not mature with time.

It is important to note that the low levels of fluoride released may be insufficient to prevent secondary caries. Krajangta *et al*. (29) reported that a fluoride concentration of at least 1 ppm is required to inhibit enamel demineralization. The materials used in this study did not fall below that range of fluoride release.

One noTable strength of this study is the use of deionized water as the storage medium. This medium is advantageous because it contains no ions, which allows for an accurate estimation of the fluoride ions released (26,28). The estimation of fluoride ions was conducted using the fluoride ion electrode method, which is a highly sensitive, specific, and rapid technique. Furthermore, the use of TISAB II buffer ensures an adequate acidic pH, which provides a direct estimation of the fluoride released (42). A further strength of this study is the measurement of flexural strength on the seventh day. It has been demonstrated that the matrix of the materials in question requires a specific period of time to mature adequately, achieving optimum properties after seven days of immersion in deionized water (31,33,38).

As a limitation, it should be noted that an *in vitro* study cannot accurately reflect the real state of the oral cavity due to its dynamic nature, which differs from laboratory conditions (46). Furthermore, the composition and pH value of saliva, plaque, and the formation of a film all have an impact on the fluoride release from restorative materials (43). The disparate methodologies employed in each study represent a significant obstacle to a meaningful comparison of results. Furthermore, assessing the flexural strength at all fluoride release times was not feasible due to the inability to use the same sample for both property measurements. The samples utilized in each test exhibit disparate measurements, thereby precluding the conduct of a comprehensive longitudinal study. Furthermore, because the test requires a fracture, we cannot use the same bar for flexural strength assessment twice.

Further clinical studies are recommended to investigate the performance of ion-releasing restorative materials and the effects on long-term flexural strength under *in vivo* environmental conditions. Furthermore, given the evidence that the materials used can absorb fluoride from topically applied gels, it is advisable to evaluate their fluoride absorption capacity (26,29,30). According to the flexural resistance found, it is recommended that the use of Ketac Universal and Equia Forte Fil be accompanied by the use of a reinforcing material.

## Conclusions

All of the ion-releasing restorative materials immersed in deionized water for seven days exhibited significant differences in flexural strength. Cention N and Ketac Universal exhibited the highest and lowest values, respectively. All ion-releasing restorative materials released fluoride at all test times, with Ketac Universal releasing the most at 1, 14, and 28 days, and Cention N releasing it at 3 and 7 days. Beautifil II demonstrated the lowest fluoride release at all evaluated times, maintaining a nearly constant release over time in comparison to the other materials. Ketac Universal and Equia Forte Fil exhibited a significant decrease in fluoride release on the third day, while Beautifil II and Cention N exhibited a significant increase in fluoride release during the same period of time.

## Figures and Tables

**Table 1:** Descriptive values and comparison of the flexural strength (MPa) of the four ion-releasing restorative materials.

Group	n	Mean	SD	SE	95% CI		Min	Max	SW	p*	Post hoc (p**)		
					LL	UL					B	C	D
A (Control)	10	19.23	2.94	0.93	17.12	21.33	15.48	23.87	0.255	<0.001*	<0.001**	<0.001**	<0.001**
B	10	82.77	5.30	1.68	78.98	86.55	72.88	89.19	0.649			<0.001**	<0.001**
C	10	97.10	4.99	1.58	93.53	100.66	89.74	103.27	0.130				<0.001**
D	10	31.38	7.68	2.43	25.88	36.87	21.08	41.28	0.154				

A: Ketac Universal (GIC), B: Beautifil II (Giomer), C: Cention N (Alkasite), D: Equia Forte Fil (HV-GIC); n: sample size; SD: Standard Deviation; SE: Standard Error of the mean; 95% CI: 95% Confidence Interval, LI: Lower Limit, UL: Upper Limit; Min: Minimum, Max: Maximum; SW: Shapiro Wilk normality test (*p*>0. 05, normal distribution); *Based on Welch’s Anova with robust one-factor intergroup variance (*p<0.05, significant differences); **Based on Tukey’s post hoc (**p<0.05, significant differences).

**Table 2:** Descriptive values and comparison of fluoride release (ppm) of each ion-releasing restorative materials over time.

Group	Time	n	Mean	SD	Median	IQR	p*	p**			
								3 days	7 days	14 days	28 days
A (Control)	1 day	10	24.69	0.94	24.45	1.78	<0.001*	<0.001**	1.000	0.047**	1.000
	3 days	10	17.46	0.79	17.35	1.48			0.047**	1.000	<0.001**
	7 days	10	18.31	0.71	18.20	1.35				1.000	0.047**
	14 days	10	17.87	0.70	17.75	1.28					<0.001**
	28 days	10	27.45	0.84	27.35	1.50					
B	1 day	10	1.76	0.27	1.74	0.55	<0.001*	0.030**	1.000	1.000	<0.001**
	3 days	10	2.06	0.26	2.07	0.52			1.000	<0.001**	1.000
	7 days	10	1.88	0.26	1.89	0.52				0.047**	0.072
	14 days	10	1.57	0.24	1.57	0.51					<0.001**
	28 days	10	2.23	0.19	2.31	0.38					
C	1 day	10	11.90	1.11	12.03	1.91	<0.001*	0.002**	0.237	1.000	1.000
	3 days	10	23.37	1.21	23.55	2.10			1.000	<0.001**	0.001**
	7 days	10	21.36	1.17	21.25	1.53				0.001	0.162
	14 days	10	11.37	0.94	11.25	1.41					1.000
	28 days	10	11.89	0.92	11.80	1.43					
D	1 day	10	15.57	0.73	15.35	1.31	<0.001*	0.047**	<0.001**	<0.001**	1.000
	3 days	10	5.16	0.43	5.24	0.69			1.000	0.047**	1.000
	7 days	10	4.89	0.43	4.89	0.59				1.000	0.047**
	14 days	10	3.35	0.47	3.37	0.60					<0.001**
	28 days	10	8.22	0.58	8.32	0.63					

A: Ketac Universal (GIC), B: Beautifil II (Giomer), C: Cention N (Alkasite), D: Equia Forte Fil (HV-GIC); n: sample size; SD: Standard Deviation; IQR: Interquartile range; *Based on Friedman’s nonparametric test (*p<0.05, significant differences); **Based on Bonferroni’s post hoc (**p<0.05, significant differences).

**Table 3:** Comparison of fluoride release (ppm) of each ion-releasing restorative materials according to time.

Group	n	1 day			3 days			7 days			14 days			28 days		
		Mean	SD	*p	Mean	SD	*p	Mean	SD	*p	Mean	SD	*p	Mean	SD	*p
A (Control)	10	24.69^a^	0.94	<0.001*	17.46^b^	0.79	<0.001*	18.31^b^	0.71	<0.001*	17.87^a^	0.70	<0.001*	27.45^a^	0.84	<0.001*
B	10	1.75^d^	0.27		2.06^d^	0.26		1.87^d^	0.26		1.56^d^	0.24		2.23^d^	0.19	
C	10	11.89^c^	1.11		23.37^a^	1.21		21.36^a^	1.17		11.36^b^	0.94		11.89^b^	0.92	
D	10	15.57^b^	0.73		5.16^c^	0.43		4.88^c^	0.43		3.34^c^	0.47		8.22^c^	0.58	

A: Ketac Universal (GIC), B: Beautifil II (Giomer), C: Cention N (Alkasite), D: Equia Forte Fil (HV-GIC); n: sample size; SD: Standard Deviation; *Based on Welch’s Anova with robust one-factor intergroup variance (*p<0.05, significant differences); a, b and c: Different letters in the mean column indicated significant differences according to Tukey’s post hoc (*p*<0.05).

## Data Availability

The datasets used and/or analyzed during the current study are available from the corresponding author.
